# Diagnostic accuracy of a rapid RT-PCR assay for point-of-care detection of influenza A/B virus at emergency department admission: A prospective evaluation during the 2017/2018 influenza season

**DOI:** 10.1371/journal.pone.0216308

**Published:** 2019-05-07

**Authors:** Maxime Maignan, Damien Viglino, Maud Hablot, Nicolas Termoz Masson, Anne Lebeugle, Roselyne Collomb Muret, Prudence Mabiala Makele, Valérie Guglielmetti, Patrice Morand, Julien Lupo, Virginie Forget, Caroline Landelle, Sylvie Larrat

**Affiliations:** 1 HP2 INSERM U1042, University Grenoble Alpes, Emergency department, Grenoble Alpes University Hospital, Grenoble, France; 2 Institut de Biologie Structurale (IBS), CEA, CNRS, University Grenoble Alpes, Laboratoire de Virologie, Grenoble Alpes University Hospital, Grenoble, France; 3 TIMC-IMAG, CNRS, Grenoble INP, University Grenoble Alpes, Infection Control Unit, Grenoble Alpes University Hospital, Grenoble, France; University of Pittsburgh, UNITED STATES

## Abstract

**Study objective:**

To investigate the performance of a rapid RT-PCR assay to detect influenza A/B at emergency department admission.

**Methods:**

This single-center prospective study recruited adult patients attending the emergency department for influenza-like illness. Triage nurses performed nasopharyngeal swab samples and ran rapid RT-PCR assays using a dedicated device (cobas Liat, Roche Diagnostics, Meylan, France) located at triage. The same swab sample was also analyzed in the department of virology using conventional RT-PCR techniques. Patients were included 24 hours-a-day, 7 days-a-week. The primary outcome was the diagnostic accuracy of the rapid RT-PCR assay performed at triage.

**Results:**

A total of 187 patients were included over 11 days in January 2018. Median age was 70 years (interquartile range 44 to 84) and 95 (51%) were male. Nine (5%) assays had to be repeated due to failure of the first assay. The sensitivity of the rapid RT-PCR assay performed at triage was 0.98 (95% confidence interval (CI): 0.91–1.00) and the specificity was 0.99 (95% CI: 0.94–1.00). A total of 92 (49%) assays were performed at night-time or during the weekend. The median time from patient entry to rapid RT-PCR assay results was 46 [interquartile range 36–55] minutes.

**Conclusion:**

Rapid RT-PCR assay performed by nurses at triage to detect influenza A/B is feasible and highly accurate.

## Introduction

Respiratory viruses are responsible for the largest annual epidemics in western countries. In the United States of America, between 30,000 and 50,000 deaths per year are directly attributable to influenza.[1] The number of emergency department visits for influenza-like illnesses is estimated at around 100 per 100 000 inhabitants.[2] The management of these epidemics is therefore a real medical challenge. Many studies have shown increased emergency department stay lengths, bed use and premature patient departures during influenza epidemics.[3–5]

During influenza epidemics, the management of patients attending the emergency department for influenza-like illness is organized around three priorities. [6] The first priority is to assess the severity of the symptoms in order to determine the most appropriate clinical pathway for the patient: discharged home, admission to a general ward or admission to an intensive care unit. The second priority is assessment of the need for antiviral treatment; this is based on factors like co-morbidities and respiratory symptom severity. Thirdly, the patient is isolated to prevent aerosol transmission of respiratory viruses.[7] Identification of the virus is therefore essential at each stage of this management to predict symptom progression and the likelihood of clinical deterioration.[8,9] Viral identification is also necessary to ensure selection of appropriate anti-viral treatments and to avoid the unnecessary use of antibiotics; this is particularly important in certain patient groups, such as those at risk of bacterial colonization.[8,10] Evaluation of the patient’s viral-status is important to determine if the patient requires isolation or not, and thus to ensure the smooth running of the emergency department and hospitalization units.[11]

The current gold-standard for the diagnosis of respiratory viruses is nucleic acid testing.[6] However, this analysis is normally performed in a virology laboratory and results are only available after several hours. New, rapid nucleic acid tests have been developed for use at point-of-care; they can be conducted at the bedside and yield results in less than 30 minutes. Laboratory studies have shown that these tests have very high sensitivity and specificity.[12–18] Rapid nucleic acid testing is simple to use and thus can be performed as soon as the patient arrives in the emergency department, allowing rapid viral identification. At the time of writing, however, data are unavailable on the diagnostic accuracy of nucleic acid testing when performed by non-specialized personnel in busy, real-world point-of-care conditions. The accuracy of such assays could be reduced by inappropriate sampling and/or incorrect use of the device. In addition, the usefulness of these assays could be questioned by operational aspects related to patient flow. The aim of this study was therefore to assess the diagnostic accuracy of a rapid RT-PCR assay for influenza A/B by nurses at a triage point of care.

## Methods

This study followed the Standards for Reporting Diagnostic Accuracy study (STARD) guidelines (Supporting information files).[19] The study was approved by both our National Review Board, the Advisory Committee on the Treatment of Information in the field of Health Research (Comité consultatif sur le traitement de l“information en matière de recherche, CCTIRS) and the national commission for liberties and data protection (Commission nationale de l“informatique et des libertés, CNIL). Informed oral consent for participation was obtained from each participant, in accordance with French law.

### Study design and setting

This study was conducted in the emergency department of a University Hospital (Grenoble Alpes University Hospital, France). At the time of writing, this hospital is the reference center for pulmonary and infectious diseases for a population of approximately 450,000 inhabitants and the Emergency Department is visited by approximately 65,000 people each year. Three other emergency departments receive low-acuity patients within the study area. Separate, dedicated facilities are available for obstetric, gynecological and pediatric emergencies.

The study took place in January 2018, when the 2017/2018 influenza season was at its peak. All patients were screened around the clock at triage, in an area that accommodated up to three patients and comprised a reception area (approximately 15m^2^) and an examination area (approximately 50m^2^). It was continuously staffed by two nurses with a third nurse from 4pm to 10pm. The nurses evaluated patients’ symptom severity, prescribed x-rays for minor limb trauma and provided analgesia, all as part of a dedicated nurse-led screening protocol. For the purposes of this study, additional nurses were not recruited nor were their tasks modified (except for Influenza testing).

### Selection of participants

Patients were eligible for participation if they were over 18 years of age, febrile (body temperature ≥ 38°C evaluated either at home or in the emergency department) and also had at least one of the following symptoms: cough, rhinorrhea, dyspnea or a sore throat. Patients with an acute exacerbation of a chronic pulmonary disease were also included. Exclusion criteria were: a clearly-identified non-respiratory infection at triage, previous identification of a virus by another means, patient currently taking oseltamivir, or contraindications for nasopharyngeal swab sampling (e.g. otorhinolaryngology neoplasia, recent facial trauma, nasal surgery or active epistaxis). Eligibility was determined by a triage nurse; a physician explained the study to the patient who was included if he or she gave oral consent for participation. Patients were included consecutively.

### Interventions

At inclusion, a nasopharyngeal swab sample was taken by a triage nurse using the Centers for Disease Control and Prevention guidelines: a flexible, flocked swab was inserted into one nostril to a depth equal to the distance between the nostrils and the outer opening of the ear.[20,21] The swab was held in place for 5 seconds and was rotated while it was slowly removed. The tip of the swab was placed into the liquid transport media inside a sterile viral sample tube (Sigma Virocult, MWE, Wiltshire, England) and the applicator stick was cut off. After 1 minute, 200 μL of this sample was taken to perform the rapid RT-PCR assay *in situ* at triage. The remainder of the sample was sent to the hospital virology laboratory for identification using the reference RT-PCR assay Human Influenza A/B PCR kit—R-DiaFlu (Diagenode Diagnostics, Ougrée, Belgium). The staff who performed the RT-PCR assay in the Virology Laboratory were unaware of the rapid RT-PCR assay results from the triage assay.

At triage, the Roche cobas influenza A/B Nucleic Acid test, a Clinical Laboratory Improvement Amendments (CLIA) waived RT-PCR assay, was used on the cobas Liat system to detect Influenza A, Influenza B, and Respiratory Syncytial Virus (RSV). One cobas Liat system was located in the triage examination area. Before the study, the triage nurses were trained to perform the nasopharyngeal swab sample and to run the rapid RT-PCR assay. The training consisted of a 10-minute video explaining the swab sampling process followed by 30 minutes of hands-on training in the use of the rapid RT-PCR assay. Following their swab, patients then either returned to the waiting room or were admitted to the emergency department monitoring area, depending on the severity of their symptoms.

All rapid assay test results were available in less than 25 minutes and were directly recorded in the patient“s electronic medical record. The physician in charge of the patient was not blind to these results since the cobas Liat system has been fully cleared by the European and French authorities, and the aim of the study was not to evaluate the impact of the results on clinical decision-making.

### Measurements

Data were prospectively collected on a dedicated case report form. The following descriptive data were recorded: age, sex, weight, height, smoking status and place of residence (home or nursing home). The Charlson Comorbidity Index was calculated.[22] Patients were asked if they had received the Influenza vaccination that year, when the symptoms began and if they had taken any antibiotics. Vital parameters at triage and the administration of any antiviral or antibiotic treatment in the emergency department were also recorded. Patient outcomes (hospitalization, intensive care unit admission and length of stay or death during hospitalization) were collected. Data relating to the emergency department activity were also collected: the number of patients presenting at the emergency department during the hour of inclusion (surrogate of triage activity) and the number of patients actually admitted to the emergency department at the time of inclusion (surrogate of emergency department activity).

### Outcomes

The aim of the study was to assess the performance characteristics of the rapid RT-PCR assay (cobas Liat system) performed at triage to detect influenza A/B, therefore RSV detection was not evaluated.

The primary outcome was the diagnostic accuracy of the rapid RT-PCR assay to detect influenza A/B, including sensitivity, specificity and the negative and positive predictive values. RT-PCR performed in the department of virology using the Human influenza A/B PCR kit—R-DiaFlu (Diagenode Diagnostics, Ougrée, Belgium) was considered as the reference value for each sample. If different results were obtained by the rapid RT-PCR assay at triage and the laboratory RT-PCR, an Xpert Xpress Flu/RSV assay (Cepheid, Sunnyvale, California, USA) was performed in the virology lab and a sample was also sent for analysis to the national influenza virus reference center (Centre National de Référence du Virus Influenza Région Sud, Lyon, France).

Secondary outcomes were the number of failures (*i*.*e*. no results because of an operational issue) of the rapid RT-PCR assay, the number of tests performed during night-shifts and at weekends, and the time between the patient’s entry into the study and obtaining the results of the rapid RT-PCR assay. Patient entry time was defined as the time that the administrative record was created; it occurred just before evaluation by the triage nurse. We also wanted to describe the activity of the triage and the emergency department when RT-PCR assays were performed.

### Statistical analysis

Based on the estimation that 45% of samples would be positive to influenza A or B, a sample size of 173 patients was required.[25] In this case, a specificity and sensitivity of 99% could be estimated with an accuracy of 2%.[23] We included at least 185 patients in order to account for possible assay failures. The performance parameter results are expressed as 95% confidence intervals (CI) and the alpha risk was set at 5%. 95% CI for sensitivity and specificity were calculated according to the score method corrected for continuity.[24,25] Categorical data are expressed as numbers and percentages and quantitative data are expressed as medians and interquartile ranges (IQR). Missing data were not replaced. Statistical analyses were performed with SPSS (v20, SPSS Inc., Chicago, IL, USA).

## Results

### Patient characteristics

**Fig 1:**
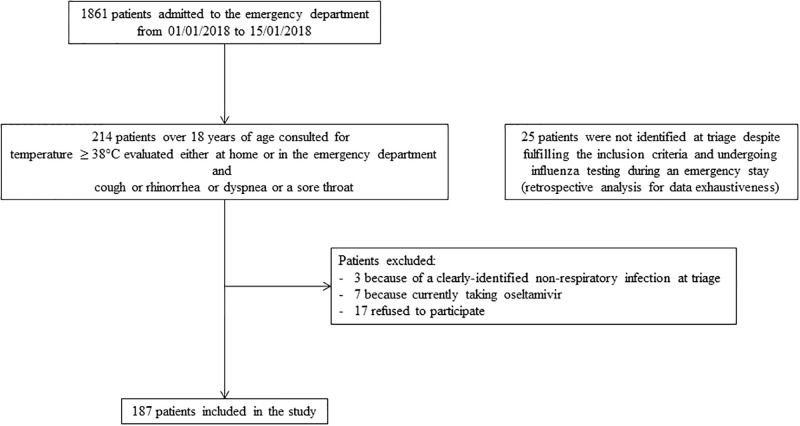
Study flow chart.

A total of 187 patients were included over the 11 days between the 4^th^ to the 15^th^ of January 2018. The study flow chart is shown in Fig 1. Patient characteristics are displayed in Table 1. Median length of stay in the emergency department was 5h 20min (Interquartile range (IQR): 4h 43min– 7h 39min). Four tests were used to calibrate the cobas Liat system. Nine tests (5%) were performed in duplicate: four because a computer network failure led to the loss of results, two because of RT-PCR failure and three for unknown reasons. Thus 200 tests were analyzed for 187 patients.

**Table 1:** Patient characteristics.

	n = 187	Missing values
Age (years)	70 [44–84]	0
Male (n, %)	95 (51)	0
Body mass index (kg/m^2^)	24.9 [21.7–27.9]	87 (47)
Charlson Comorbidity Index	2 [0–3]	11 (6)
Living in nursing home	25 (13)	0
Smoking status		47 (25)
Current	15 (8)	
Previous	59 (32)	
Influenza vaccination (yes)	40 (21)	83 (44)
Time since symptom onset (days)	3 [1–7]	18 (10)
Antibiotic treatment prior to ED visit	39 (21)	38 (20)
Vital parameters at triage		
Temperature (°C)	37.4 [36.9–38.3]	0
Oxygen saturation (%)	96 [93–98]	1 (0.5)
Respiratory rate (/min)	22 [17–30]	1 (0.5)
Heart rate (/min)	91 [79–102]	0
Systolic arterial pressure (mmHg)	135 [118–156]	0
Glasgow coma scale	15 [15–15]	0
Outcomes		
Hospitalization	85 (45)	0
Length of hospitalization (days)	6 [4–9]	0
Intensive care unit admission	6 (3)	0
Death	11 (6)	0

Data are presented as n (%) or median [interquartile]. ED: emergency department

### Sensitivity and specificity

The rapid RT-PCR assay at triage identified a viral infection in 97 (52%) patients: 28 (15%) patients had a positive result for influenza A, 44 (24%) patients had a positive result for influenza B and 25 (13%) patients had a positive result for RSV. No viral co-infections were detected. The sensitivity and specificity of the rapid RT-PCR assay at triage to detect Influenza viruses were very high and only three results from the rapid RT-PCR assay at triage were different from the laboratory assay results. Of these three, two positive results for influenza A at triage were found to be negative in the department of virology. Further analysis of two of these samples, using Xpert Xpress Flu/RSV assay found one positive and one negative result, findings that were confirmed by the national reference center. The third result discrepancy was a negative influenza B triage result that was found to be positive by both assays in the department of virology and by the national reference center. Thus the rapid RT-PCR assay at triage had a global sensitivity for influenza A/B of 0.98 (95% CI: 0.91–1.00) and a specificity of 0.99 (95% CI: 0.94–1.00). The negative predictive value of the rapid PCR assay was 0.99 (95% CI: 0.94–1.00) and the positive predictive value was 0.99 (0.91–1.00). The results are shown in Table 2.

**Table 2:** Performance characteristics of the RT-PCR assay performed at triage.

	Influenza A/ B		Influenza A		Influenza B	
Rapid RT-PCR assay in the ED	Positive	Negative	Positive	Negative	Positive	Negative
Positive (n)	71	1	27	1	44	0
Negative (n)	1	114	0	159	1	142
Sensitivity	0.98 (0.91–1.00)		1.00 (0.84–1.00)		0.98 (0.87–1.00)	
Specificity	0.99 (0.94–1.00)		0.99 (0.96–1.00)		1.00 (0.97–1.00)	
NPV	0.99 (0.94–1.00)		1.00 (0.97–1.00)		0.99 (0.96–1.00)	
PPV	0.98 (0.91–1.00)		0.96 (0.79–1.00)		1.00 (0.90–1.00)	

Data are presented as n or rate (95% CI). ED: emergency department; NPV: negative predictive value; PPV: positive predictive value

### Emergency department activity

A total of 67 (36%) rapid RT-PCR assays were performed during the night and 25 (13%) at weekends. Median time from patient entry to rapid RT-PCR assay results was 46 (IQR: 36–55) minutes. The median number of patients admitted during the hour in which each RT-PCR assay was performed was 10 (IQR: 7–11). During the study period, the median number of patients presenting per hour was 7 (IQR: 6–7). The median total number of patients present in the emergency department while each RT-PCR assay was performed was 52 (IQR: 44–60). During the study period, the median number of patients in the emergency department was 38 (IQR: 35–42).

## Discussion

This study demonstrated that the detection of influenza A/B virus using rapid RT-PCR assay at triage performed by nurses 24 hours-a-day, 7 days-a-week was both feasible and reliable. The sensitivity and specificity of the cobas Liat system for the detection of influenza A/B was higher than 0.98, even when performed by a trained professional (in this case a triage nurse) without direct laboratory supervision. Less than five percent of tests failed.

Several other studies evaluated the accuracy of RT-PCR assay in the emergency department. One study, that used the cobas Liat system in four emergency departments and eight outpatient clinics, reported a sensitivity and specificity above 0.97.[18] Two other studies that used the Cepheid“s GeneXpert Xpert Flu assay in a population of adults presenting to the emergency department reported a sensitivity of 0.95 and a specificity of 0.99.[26,27] The Alere Influenza A&B (now ID NOW) has also been tested in an emergency department setting, and showed good specificity (0.98) but low sensitivity (0.64).[28,29] Direct comparison of rapid molecular tests for the detection of influenza was carried out under laboratory conditions using samples from patients admitted to an emergency department. In those conditions, the sensitivities of the Abbott, Roche, and Cepheid tests were 0.91, 0.96, and 0.97 respectively and the overall specificities were 0.99, 0.98, and 0.98 respectively.[30] However, in all these studies, the tests were performed by laboratory assistants not healthcare professionals in real-life care settings. Furthermore, it is unclear whether tests were all performed in the ED or in laboratory of virology. Only large emergency centers can afford to employ a laboratory assistant [31] and this means that nucleic acid testing is usually performed either in the virology department by a laboratory assistant, which automatically lengthens the delay in obtaining the results, or in the emergency department by a clinician who is less skilled in the analysis of biological samples.

Only a few studies that evaluated RT-PCR assays respected the constraints related to use in an emergency department. Two studies compared assays from the cobas Liat system performed in the emergency department to Cepheid“s GeneXpert Xpert Flu assays (rapid central testing) during the 2017–2018 flu season. [32,33] The sensitivity and specificity of the cobas Liat system ranged from 0.85 to 0.99 and 0.96 to 0.99, respectively. The study that reported the lower sensitivity might have been subject to bias because only negative results were systematically centrally controlled, thus increasing the likelihood of false positives. Only one study investigated the performance of RT-PCR assay at triage [11] and found a sensitivity and specificity of respectively 1.00 and 0.95. However, this result is based on only 38 specimens obtained during a normal working day. Furthermore the samples used were only incidentally assayed using the rapid RT-PCR as well as the laboratory RT-PCR test, and thus results were at risk of being biased because it was not clear why they were selected to be tested twice.

The ease of performing rapid RT-PCR assays at the triage point of care and the accuracy of the results meant that the early viral identification facilitated appropriate treatment more quickly than if laboratory viral analysis had been used. In previous studies which failed to show an effect, or showed a minor effect, of nucleic acid testing on clinical decision-making in patients with respiratory symptoms it is notable that many clinical decisions, especially involving antibiotic prescriptions, were made before the viral identification test results were received.[34] Although triage is a complex area in the emergency department, with very specific constraints for staff, early nucleic acid testing would allow informed clinical decisions to be made. In the present study, very few tests failed, despite the fact 49% were performed at night or weekends when staffing levels are routinely lower. Even though the majority of RT-PCR assays were performed during the busiest hours for the emergency department, results were available within a median of 46 minutes from a patient’s entry into triage. Such a quick result would allow prompt decisions.[7,35] Some authors have shown that the use of rapid RT-PCR assays in the emergency department may shorten patient length of stay by at least one hour.[28,32,36,37]

It must be remembered that identification of the virus is only a part of the clinical diagnosis and management. Although the results of this study are important, further studies are necessary to assess the clinical impact of influenza RT-PCR assay at triage in order to evaluate its effects on decision-making and the prescription of complementary examinations. Point of care testing is only useful if it has an impact on treatment. Some studies have shown a positive impact on medical costs [36,38] and antiviral treatment [37], especially for patients with an influenza negative diagnosis, highlighting the importance of tests that have a high negative predictive value. Interestingly, one study demonstrated a change in clinical prescribing practices when using a point-of-care RT-PCR test compared with a rapid antigen detection, suggesting that clinicians had greater confidence in those tests.[39] However, a rapid diagnosis not only allows antiviral treatment to be administered early, but also prevents such treatment being administered unnecessarily. For example, it could prevent the unnecessary prescription of drugs such as neuraminidase inhibitors (oseltamivir, zanamivir), which are recommended for patients with severe respiratory symptoms and limit contagion, however they have also been found to lead to drug resistance.[40,41] Finally, future studies should also investigate the performance of the cobas Liat system for the detection of RSV.

This study had several limitations. First, the percentage of positive results for influenza was lower than that anticipated in the sample size calculation (39% vs. 45%). As a result, the accuracy of the estimation of sensitivity and specificity was lower than expected. Second, this study was conducted during an epidemic peak and so the results should be viewed in the context of the conditions during such a period. Positive predictive values decrease when tests are performed with a lower prevalence of the disease, thus the results cannot be extrapolated to a lower prevalence period. Moreover, it must be noted that the results may differ slightly depending on influenza strains and epidemics. Third, this study was conducted in a single care center setting. The results may therefore be specific to the population of this center and may not necessarily be applicable to other patient groups or settings such as children or smaller facilities, for example. Use of the rapid RT-PCR assay at triage in other care settings would depend on the organization of each emergency department and, importantly, on the availability of appropriately trained staff. Fourth, high rates of missing values are reported in Table 1. This may be partly explained by the lack of a dedicated research nurse or clinical research assistant at triage. Case report forms were completed by the triage nurse under the indirect supervision of a research nurse. This was deliberate in order to evaluate real-world conditions. Finally, patients without fever either at home or on admission to the emergency department were not included. Not all patients who actually have influenza have fever, particularly older people, which may have led to sampling and spectrum bias.[42] Nevertheless, this criterion is very commonly used in studies investigating the performance of novel PCR assay to diagnose influenza.[11,32] Moreover, we decided to include patients if they reported fever at home even if body temperature was normal at inclusion. This decision was made to consider a possible treatment-induced decrease in fever (e.g. paracetamol intake during the hours prior to emergency department admission).

## Conclusion

The diagnosis of respiratory viruses by rapid point of care nucleic acid tests carried out in the emergency department, rather than being sent away to a laboratory, has the potential to dramatically improve patient care during seasonal influenza epidemics. This study demonstrated the feasibility and the high accuracy of rapid RT-PCR assay to detect influenza A/B when performed by trained nurses at triage. Further studies are now needed to determine the impact of such tests on clinical decision making and patient outcomes.

## Supporting information

S1 File List of essential items for reporting diagnostic accuracy studies. STARD 2015.
(DOCX)

S2 File Anonymized data of the study. (PDF)
