# Supplementary material for: Diagnostic accuracy of a rapid RT-PCR assay for point-of-care detection of influenza A/B virus at emergency department admission: A prospective evaluation during the 2017/2018 influenza season
Source: PLoS One. 2019 May 7;14(5):e0216308. doi: 10.1371/journal.pone.0216308 (PMC6504036; doi:10.1371/journal.pone.0216308)
Supplement: S2 File — (PDF) [file pone.0216308.s002.pdf]

num_inclusion	lenght_stay_ED	age	gender	BMI	institution	charlson
10	07:52:00	31,00	0,00	19,59	0,00	0,00
13	07:49:00	91,00	1,00	22,22	0,00	2,00
11	01:31:00	44,00	0,00		0,00	0,00
12	04:15:00	87,00	0,00	25,35	0,00	2,00
15	02:04:00	55,00	1,00	36,11	0,00	1,00
14	10:23:00	101,00	0,00		0,00	0,00
16	08:15:00	95,00	1,00	22,89	1,00	5,00
17	04:05:00	82,00	0,00		0,00	2,00
145	11:10:00	81,00	0,00	22,95	0,00	3,00
18	06:48:00	84,00	0,00		0,00	4,00
19	05:58:00	80,00	1,00	30,85	0,00	0,00
150	07:33:00	73,00	0,00	37,98	0,00	7,00
90	05:14:00	89,00	1,00	31,98	0,00	5,00
89	06:44:00	89,00	1,00		0,00	6,00
86	02:07:00	89,00	1,00	21,22	0,00	
29	01:36:00	20,00	1,00		0,00	0,00
87	04:11:00	65,00	1,00	27,34	0,00	3,00
79	05:27:00	72,00	0,00	24,22	0,00	3,00
138	08:30:00	24,00	1,00		0,00	0,00
181	07:54:00	47,00	1,00		0,00	0,00
9	02:36:00	26,00	0,00	28,40	0,00	0,00
6	11:51:00	53,00	1,00	21,22	0,00	1,00
3	22:24:00	68,00	0,00	26,54	0,00	2,00
113	01:55:00	42,00	1,00		0,00	0,00
4	04:36:00	87,00	0,00	24,22	1,00	5,00
1	07:33:00	49,00	0,00		0,00	2,00
2	07:34:00	75,00	1,00	19,07	0,00	3,00
110	05:57:00	78,00	0,00		0,00	2,00
171	06:27:00	78,00	0,00		0,00	0,00
163	01:14:00	27,00	0,00		0,00	0,00
76	06:01:00	94,00	1,00	44,19	0,00	3,00
136	03:33:00	28,00	1,00		0,00	0,00
75	07:50:00	30,00	1,00	27,17	1,00	0,00
119	05:10:00	47,00	0,00		0,00	0,00
132	05:23:00	62,00	1,00		0,00	1,00
77	00:34:00	26,00	0,00		0,00	0,00
159	04:24:00	87,00	1,00	14,83	0,00	2,00
96	04:40:00	66,00	0,00	21,55	0,00	
100	06:38:00	79,00	0,00	20,76	1,00	11,00
98	04:14:00	20,00	1,00		0,00	0,00
95	06:57:00	82,00	0,00	30,85	0,00	3,00
186	05:05:00	87,00	1,00		1,00	2,00
94	03:18:00	91,00	0,00	15,67	1,00	3,00
97	01:55:00	55,00	1,00	21,37	0,00	0,00
170	02:31:00	25,00	0,00		0,00	
92	09:34:00	30,00	1,00	19,92	0,00	0,00
118	07:32:00	24,00	0,00		0,00	0,00
126	06:17:00	73,00	1,00		0,00	1,00
127	02:19:00	95,00	1,00		0,00	0,00

111	03:25:00	44,00	1,00		0,00	2,00
39	07:21:00	54,00	1,00	23,44	0,00	2,00
38	07:19:00	89,00	0,00	24,22	0,00	6,00
42	09:06:00	87,00	0,00	20,18	0,00	
177	23:47:00	45,00	1,00		0,00	1,00
116	14:28:00	84,00	0,00		0,00	4,00
41	06:07:00	65,00	1,00	37,89	0,00	0,00
40	10:03:00	84,00	1,00	23,44	0,00	2,00
187	04:57:00	72,00	0,00	25,91	0,00	7,00
43	04:41:00	50,00	1,00	26,42	0,00	0,00
148	00:46:00	87,00	1,00		0,00	7,00
34	07:14:00	19,00	1,00	26,95	0,00	0,00
35	04:44:00	23,00	1,00		0,00	0,00
121	05:39:00	70,00	1,00		0,00	2,00
133	07:41:00	79,00	0,00		0,00	5,00
153	06:00:00	87,00	1,00	21,63	0,00	1,00
176	07:39:00	81,00	1,00		0,00	3,00
158	05:20:00	40,00	1,00	19,57	0,00	1,00
184	01:32:00	39,00	0,00		0,00	
88	04:53:00	65,00	1,00	27,34	0,00	2,00
78	02:34:00	97,00	0,00		0,00	
32	23:01:00	27,00	1,00	19,15	0,00	0,00
36	05:46:00	62,00	0,00		0,00	1,00
37	13:55:00	88,00	1,00		1,00	1,00
31	12:30:00	79,00	0,00	27,34	0,00	
33	05:38:00	23,00	1,00	26,12	0,00	0,00
81	04:39:00	36,00	0,00	27,78	0,00	0,00
30	06:02:00	95,00	1,00		1,00	2,00
80	03:39:00	80,00	1,00	29,30	0,00	3,00
84	04:13:00	86,00	1,00		1,00	6,00
82	05:14:00	78,00	0,00	27,76	0,00	1,00
83	07:07:00	52,00	0,00	24,68	0,00	0,00
85	05:51:00	71,00	0,00		0,00	1,00
185	05:31:00	78,00	1,00	23,96	0,00	9,00
108	09:35:00	77,00	0,00		0,00	3,00
147	14:01:00	64,00	0,00		1,00	5,00
144	15:36:00	78,00	0,00	24,17	0,00	3,00
28	07:47:00	64,00	1,00		0,00	0,00
27	00:50:00	50,00	1,00		0,00	0,00
25	06:04:00	67,00	1,00		0,00	5,00
26	09:30:00	81,00	0,00	28,40	0,00	2,00
24	08:09:00	71,00	1,00		0,00	3,00
169	05:10:00	81,00	0,00		0,00	2,00
23	07:01:00	90,00	1,00	34,17	0,00	1,00
114	16:37:00	95,00	1,00		1,00	2,00
66	23:26:00	89,00	1,00	27,06	1,00	4,00
59	20:20:00	92,00	1,00		0,00	
141	19:50:00	84,00	0,00	28,13	0,00	0,00
115	05:00:00	53,00	1,00		0,00	1,00
22	06:05:00	89,00	1,00	21,23	0,00	

61	18:01:00	55,00	1,00	41,44	0,00	9,00
21	04:40:00	40,00	0,00	31,18	0,00	0,00
54	18:00:00	69,00	0,00	23,74	0,00	
20	06:13:00	97,00	1,00	27,89	1,00	2,00
60	18:37:00	84,00	1,00		0,00	4,00
63	14:55:00	87,00	0,00	34,60	1,00	3,00
57	14:48:00	79,00	0,00	24,21	0,00	5,00
58	04:23:00	65,00	1,00		0,00	1,00
62	04:58:00	20,00	0,00		0,00	0,00
51	13:09:00	38,00	0,00	25,76	1,00	0,00
64	13:34:00	85,00	1,00	25,78	0,00	0,00
50	15:19:00	86,00	0,00	20,83	1,00	
53	12:30:00	82,00	0,00	29,76	0,00	4,00
56	09:56:00	51,00	0,00	39,84	0,00	3,00
154	07:00:00	64,00	0,00	21,19	0,00	4,00
47	06:14:00	84,00	0,00	22,13	0,00	2,00
52	04:46:00	84,00	1,00		0,00	0,00
134	07:54:00	33,00	0,00		0,00	0,00
46	06:15:00	60,00	0,00	24,69	0,00	6,00
49	08:19:00	61,00	1,00		0,00	0,00
122	07:35:00	23,00	1,00		0,00	0,00
48	01:51:00	38,00	1,00	27,68	0,00	0,00
149	08:18:00	88,00	1,00		0,00	5,00
125	02:42:00	22,00	0,00		0,00	0,00
183	18:56:00	70,00	1,00	27,81	0,00	8,00
180	09:53:00	25,00	0,00		0,00	0,00
157	10:10:00	76,00	1,00	19,34	0,00	2,00
142	01:47:00	40,00	1,00		0,00	0,00
137	09:21:00	53,00	0,00	25,26	0,00	2,00
45	03:29:00	31,00	0,00		0,00	0,00
117	06:29:00	86,00	0,00		0,00	2,00
70	18:03:00	33,00	0,00	27,78	0,00	0,00
73	01:55:00	59,00	1,00	32,42	1,00	3,00
65	01:43:00	27,00	1,00		0,00	0,00
140	09:29:00	73,00	0,00		1,00	0,00
69	09:54:00	88,00	1,00		0,00	10,00
152	05:11:00	37,00	1,00		0,00	0,00
104	03:45:00	82,00	0,00		0,00	
71	23:45:00	22,00	1,00	22,58	0,00	0,00
68	08:10:00	85,00	0,00		0,00	5,00
146	07:57:00	95,00	0,00		1,00	0,00
139	07:14:00	81,00	0,00	21,88	0,00	1,00
179	04:20:00	41,00	1,00		0,00	0,00
175	21:20:00	83,00	1,00	19,68	0,00	5,00
172	12:38:00	97,00	1,00		1,00	3,00
130	07:55:00	51,00	1,00		0,00	0,00
72	14:59:00	86,00	0,00	25,95	1,00	6,00
99	05:18:00	83,00	1,00		0,00	
74	00:38:00	23,00	0,00	30,42	1,00	1,00
55	09:19:00	78,00	0,00	24,91	0,00	4,00

67	00:10:00	49,00	1,00	21,89	0,00	0,00
102	06:24:00	70,00	0,00		0,00	0,00
101	04:51:00	77,00	0,00	21,79	0,00	2,00
174	05:11:00	42,00	1,00		0,00	0,00
103	07:04:00	81,00	1,00	19,98	0,00	3,00
167	03:22:00	87,00	1,00	24,89	1,00	3,00
151	10:20:00	74,00	0,00	24,21	0,00	1,00
106	08:34:00	87,00	0,00	24,15	0,00	4,00
173	09:05:00	54,00	1,00		0,00	0,00
166	07:12:00	88,00	0,00	20,02	1,00	3,00
129	08:32:00	88,00	1,00	29,75	0,00	3,00
93	22:18:00	86,00	0,00		1,00	2,00
143	19:51:00	90,00	1,00	25,59	0,00	1,00
161	05:30:00	82,00	1,00	31,04	0,00	2,00
109	18:43:00	57,00	0,00		0,00	8,00
105	07:28:00	25,00	1,00	18,59	0,00	0,00
182	06:17:00	37,00	0,00		0,00	2,00
107	04:45:00	48,00	1,00		0,00	1,00
135	02:49:00	17,00	0,00		0,00	1,00
164	05:30:00	69,00	0,00		0,00	0,00
162	05:34:00	90,00	1,00	20,96	0,00	4,00
7	08:11:00	76,00	0,00		0,00	6,00
155	17:40:00	51,00	1,00	24,61	0,00	2,00
178	05:08:00	89,00	1,00	22,98	0,00	3,00
165	03:29:00	44,00	0,00		0,00	
123	03:17:00	39,00	0,00		0,00	0,00
124	09:16:00	95,00	1,00		0,00	4,00
120	12:21:00	65,00	1,00		0,00	3,00
8	23:29:00	57,00	0,00		0,00	2,00
168	08:10:00	83,00	0,00	25,62	0,00	3,00
156	02:11:00	21,00	0,00		0,00	0,00
5	05:14:00	48,00	0,00	35,92	0,00	0,00
131	03:56:00	44,00	0,00		0,00	0,00
112	01:08:00	24,00	0,00		0,00	0,00
44	10:32:00	78,00	0,00	29,40	0,00	
128	04:03:00	63,00	0,00		0,00	2,00
160	08:12:00	64,00	0,00	34,31	0,00	4,00
91	00:41:00	35,00	1,00	20,76	0,00	0,00

ctive_smokin	past_smoking	ntibiotic_pric	vaccin_flu	delay_sympt	resp_rate	temp	saturation
1,00	1,00	0,00	2,00	2,00		36,00	96,00
0,00	0,00	0,00	0,00	5,00		37,40	98,00
0,00	0,00	1,00	0,00	4,00	15,00	37,00	96,00
0,00	1,00	1,00	1,00	7,00	20,00	37,10	97,00
0,00	0,00	0,00	0,00	2,00	19,00	38,00	94,00
		0,00		3,00		38,50	97,00
0,00	2,00	0,00	2,00	1,00	32,00	36,40	94,00
0,00	1,00	1,00	2,00	1,00		37,20	98,00
0,00	1,00	0,00		4,00	36,00	38,50	93,00
0,00	1,00	1,00	0,00	3,00	19,00	37,40	90,00
0,00	0,00	1,00	2,00	7,00		36,40	95,00
0,00	1,00	0,00	1,00	15,00		37,60	86,00
0,00	0,00	0,00	0,00	3,00	20,00	37,10	97,00
0,00	2,00	0,00	1,00	1,00	30,00	37,40	98,00
0,00	0,00	0,00	0,00	3,00	18,00	37,50	95,00
0,00	0,00	0,00	2,00	10,00		36,60	100,00
0,00	1,00	1,00	1,00	8,00	16,00	37,00	95,00
0,00	1,00	1,00	2,00	7,00	20,00	37,20	95,00
0,00	0,00	0,00	0,00	1,00		38,20	99,00
						38,20	97,00
0,00	0,00	0,00	0,00	1,00	0,00	37,10	
1,00	1,00	1,00	0,00	3,00	25,00	37,10	94,00
1,00	0,00	0,00	1,00	2,00	25,00	37,10	97,00
1,00	1,00	0,00		15,00		36,00	98,00
0,00	0,00	0,00	2,00	1,00	30,00	37,10	88,00
1,00	1,00	0,00	0,00	1,00	20,00	37,00	96,00
0,00	0,00	0,00	0,00	15,00	20,00	36,80	94,00
		0,00		1,00		39,90	90,00
				2,00		37,50	98,00
0,00	0,00	0,00	0,00	15,00		37,10	96,00
0,00	0,00	1,00	1,00	4,00	38,00	39,20	92,00
1,00	1,00	0,00		15,00		37,10	99,00
0,00	0,00	0,00	0,00	1,00	14,00	37,80	99,00
1,00	1,00	0,00		1,00		37,80	96,00
		0,00		15,00	30,00	37,70	92,00
0,00	2,00	0,00	0,00	3,00	15,00	37,70	95,00
		0,00	1,00	1,00		39,20	97,00
0,00	0,00	0,00	0,00	15,00	9,00	36,40	94,00
0,00	1,00	1,00	2,00	7,00	20,00	36,90	97,00
0,00	0,00	0,00	0,00	1,00	12,00	38,50	100,00
0,00	0,00		0,00	5,00	30,00	36,70	100,00
				1,00		39,00	92,00
0,00	0,00	1,00	1,00	5,00	27,00	37,90	95,00
1,00	1,00	0,00	0,00	2,00		39,40	
				2,00		39,60	98,00
0,00	0,00	0,00	0,00	10,00	15,00	37,50	98,00
0,00	0,00	0,00		2,00		39,20	97,00
		0,00		1,00		38,40	98,00
		0,00		1,00		37,00	93,00

	1,00	0,00		1,00		36,90	95,00
1,00	1,00	0,00	2,00	1,00	30,00	37,00	99,00
2,00	2,00	0,00	2,00	3,00	20,00	36,90	98,00
0,00	0,00	1,00		14,00	30,00	38,50	96,00
				4,00		37,20	96,00
		0,00		7,00	30,00	37,90	95,00
1,00	1,00	0,00	2,00	7,00	16,00	37,20	95,00
0,00	0,00	1,00	1,00	15,00	38,00	39,00	80,00
0,00	1,00	0,00		7,00		36,80	93,00
0,00	0,00	0,00	0,00	5,00	17,00	38,50	98,00
		1,00				36,60	90,00
1,00	1,00	0,00	0,00	10,00	12,00	37,30	99,00
0,00	0,00	0,00	0,00	1,00		38,10	100,00
0,00	1,00	1,00		5,00		37,40	95,00
0,00	1,00	0,00		2,00		36,60	96,00
		0,00	1,00	2,00		37,30	97,00
				5,00		38,10	99,00
				2,00		37,00	96,00
1,00	0,00		1,00	15,00			
0,00	0,00	0,00	1,00	7,00	15,00	36,70	100,00
0,00	0,00	0,00	2,00	2,00	26,00	36,90	94,00
0,00	0,00	0,00	0,00	8,00	32,00	38,40	94,00
0,00	0,00	0,00	0,00	3,00	32,00	38,40	92,00
0,00	0,00	1,00	2,00	2,00	36,00	37,80	94,00
0,00	1,00	0,00	1,00	1,00	32,00	38,40	93,00
0,00	0,00	0,00	0,00	8,00	20,00	37,00	98,00
1,00	1,00	0,00	0,00	6,00	25,00	36,90	97,00
2,00	2,00	1,00	2,00	3,00	29,00	36,60	93,00
0,00	0,00	0,00	0,00	1,00	15,00	38,20	79,00
0,00	0,00	1,00	2,00	11,00		38,40	95,00
0,00	1,00	1,00	0,00	8,00	15,00	37,60	97,00
1,00	1,00	0,00	2,00	1,00	15,00	37,80	96,00
0,00	0,00	0,00	0,00	15,00		38,90	94,00
0,00	0,00	0,00	0,00	1,00		39,20	91,00
		0,00				37,50	96,00
		0,00				38,30	93,00
0,00	1,00	0,00		1,00		37,30	94,00
1,00	1,00	0,00	0,00	3,00		37,80	96,00
0,00	2,00	0,00	0,00	2,00		37,40	96,00
0,00	1,00	0,00	1,00	3,00	33,00	36,00	92,00
0,00	1,00	0,00	1,00	7,00	17,00	36,80	93,00
1,00	1,00	0,00	0,00	1,00		39,40	98,00
				3,00		39,20	96,00
0,00	2,00	1,00	2,00	10,00	30,00	36,10	91,00
0,00	0,00	1,00	1,00	12,00	30,00	37,00	97,00
0,00	0,00	1,00	1,00	5,00	24,00	38,80	87,00
2,00	2,00	2,00	1,00	1,00		38,80	93,00
0,00	0,00	0,00	0,00	4,00		35,60	95,00
1,00	1,00	0,00		1,00		36,60	96,00
0,00	1,00	0,00	1,00	3,00	35,00	37,00	95,00

0,00	0,00	0,00	0,00		20,00	36,90	95,00
1,00	1,00	0,00	0,00	4,00	26,00	37,70	91,00
0,00	1,00	1,00	1,00	2,00	22,00	36,90	93,00
0,00	0,00	0,00	2,00	1,00	26,00	38,60	92,00
				1,00	25,00	36,40	95,00
0,00	1,00	0,00	2,00	1,00	26,00	37,40	89,00
0,00	1,00	0,00	2,00	2,00		37,50	98,00
				1,00	20,00	38,60	98,00
1,00	1,00	0,00	0,00	2,00		37,20	99,00
0,00	1,00	0,00	0,00	3,00	24,00	40,00	92,00
0,00	0,00	1,00	0,00	3,00	25,00	38,90	87,00
0,00	0,00	0,00	2,00	1,00	32,00	36,50	87,00
0,00	1,00	0,00	1,00	1,00	30,00	39,10	99,00
0,00	0,00	0,00	0,00	7,00	29,00	38,50	95,00
1,00	0,00	0,00		10,00		38,40	97,00
0,00	1,00	0,00	0,00	2,00	15,00	39,10	98,00
0,00	0,00	1,00	0,00	10,00	28,00	36,90	96,00
				15,00		36,60	97,00
0,00	0,00	0,00	0,00	1,00	15,00	38,00	96,00
0,00	2,00	0,00	2,00	2,00		39,10	95,00
		1,00		5,00	18,00	37,10	99,00
0,00	0,00	0,00	0,00	1,00		39,10	98,00
0,00		0,00				36,60	98,00
0,00	0,00	1,00		3,00		36,90	99,00
				10,00		37,60	77,00
0,00	0,00	0,00				37,50	95,00
0,00	0,00	0,00		1,00		39,00	98,00
		0,00		7,00		37,60	96,00
1,00	1,00	0,00	1,00		38,00	35,70	94,00
2,00	2,00	0,00	0,00	2,00		39,50	96,00
		1,00		2,00		35,90	94,00
0,00	0,00	0,00	0,00	10,00	12,00	37,10	99,00
0,00	0,00	0,00	0,00	10,00	15,00	36,60	100,00
0,00	0,00	1,00	0,00	8,00	15,00	37,20	100,00
		0,00		1,00		38,50	96,00
0,00	2,00	1,00	0,00	10,00	22,00	36,10	93,00
0,00		0,00		1,00		37,30	99,00
0,00	1,00	0,00	1,00	1,00		38,30	97,00
0,00	1,00	0,00	0,00	2,00	17,00	38,90	100,00
0,00	1,00	2,00	2,00	1,00		36,90	99,00
		0,00	0,00	1,00	24,00	37,90	97,00
0,00	1,00	0,00				37,30	97,00
1,00	0,00	0,00		15,00		36,40	99,00
						36,70	92,00
						37,40	82,00
		0,00		2,00		38,10	100,00
0,00	1,00	0,00	1,00	2,00	20,00	38,00	90,00
0,00	2,00	1,00	1,00	8,00	30,00	35,70	95,00
0,00	1,00	0,00	0,00	1,00	18,00	38,20	100,00
0,00	2,00	0,00	2,00	8,00	26,00	37,00	98,00

0,00	0,00	1,00	1,00	2,00	15,00	36,40	98,00
2,00	2,00	0,00	2,00	3,00		37,10	96,00
0,00	1,00	1,00	0,00	12,00	22,00	37,20	88,00
						37,10	99,00
0,00	0,00	0,00	1,00	1,00	40,00	36,60	99,00
0,00	1,00	0,00	0,00		36,00	36,00	96,00
		0,00	1,00			37,60	95,00
0,00	1,00	0,00	0,00	1,00	17,00	38,20	97,00
						36,30	92,00
0,00	1,00	0,00				37,50	98,00
0,00	0,00	1,00		4,00		36,70	96,00
0,00	2,00	0,00	2,00	0,00	34,00	38,00	97,00
0,00	0,00	0,00	0,00	1,00		38,10	98,00
		0,00		10,00		37,10	91,00
0,00	1,00	0,00		1,00		38,50	93,00
0,00	0,00	1,00	0,00	3,00	17,00	36,70	98,00
				4,00		37,50	90,00
0,00	0,00	0,00		6,00	22,00	36,10	98,00
		0,00		1,00		36,90	100,00
					21,00	36,40	100,00
				21,00		36,70	92,00
0,00	1,00	0,00	1,00	3,00	20,00	37,70	96,00
		0,00		3,00		37,00	97,00
0,00	1,00	0,00			20,00	37,90	92,00
						37,80	93,00
		0,00		5,00		38,20	96,00
		0,00		1,00	24,00	37,30	92,00
0,00	1,00	0,00		1,00		38,90	96,00
		0,00	0,00	7,00	20,00	39,70	84,00
					19,00	39,60	96,00
0,00	0,00	0,00		2,00		38,70	97,00
1,00	1,00	0,00	0,00	4,00	15,00	38,60	93,00
					16,00	38,60	95,00
		0,00				37,60	99,00
	1,00	0,00	1,00	1,00	38,00	38,10	100,00
0,00	1,00	0,00	0,00	2,00	37,00	38,90	93,00
		1,00	1,00	3,00		37,10	97,00
0,00	0,00	0,00	0,00	3,00	15,00	37,00	100,00

oxygene	heart_rate	stolic_pressu	glasgow	fluA	fluB	RSV	lab_fluA
0,00	80,00	100,00	15,00	1,00	0,00	0,00	1,00
0,00	97,00	159,00	15,00	0,00	0,00	0,00	0,00
0,00	88,00	116,00	15,00	0,00	1,00	0,00	0,00
0,00	74,00	88,00	15,00	0,00	1,00	0,00	0,00
0,00	112,00	131,00	15,00	0,00	0,00	0,00	0,00
0,00	83,00	176,00	15,00	0,00	0,00	0,00	0,00
0,00	56,00	151,00	15,00	0,00	0,00	1,00	0,00
0,00	116,00	155,00	15,00	0,00	0,00	0,00	0,00
9,00	81,00	146,00	15,00	0,00	0,00	0,00	0,00
4,00	68,00	98,00	15,00	0,00	1,00	0,00	0,00
0,00	72,00	152,00	15,00	0,00	0,00	0,00	0,00
5,00	72,00	172,00	15,00	0,00	1,00	0,00	0,00
0,00	86,00	137,00	15,00	0,00	1,00	0,00	0,00
0,00	96,00	149,00	15,00	0,00	0,00	0,00	0,00
0,00	57,00	203,00	15,00	0,00	0,00	0,00	0,00
0,00	94,00	129,00	15,00	0,00	0,00	0,00	0,00
0,00	86,00	114,00	15,00	0,00	0,00	0,00	0,00
0,00	96,00	177,00	15,00	0,00	0,00	0,00	0,00
0,00	91,00	140,00	15,00	0,00	0,00	0,00	0,00
0,00	118,00	156,00	15,00	0,00	1,00	0,00	0,00
0,00	135,00	70,00	15,00	1,00	0,00	0,00	0,00
2,00	102,00	146,00	15,00	0,00	0,00	1,00	0,00
0,00	89,00	123,00	15,00	0,00	0,00	0,00	0,00
0,00	87,00	138,00	15,00	0,00	1,00	0,00	0,00
15,00	130,00	133,00	3,00	0,00	0,00	0,00	0,00
0,00	86,00	113,00	15,00	0,00	0,00	0,00	0,00
0,00	60,00	200,00	15,00	0,00	0,00	1,00	0,00
0,00	93,00	188,00	15,00	0,00	1,00	0,00	0,00
0,00	75,00	171,00	15,00	0,00	0,00	0,00	0,00
0,00	120,00	111,00	15,00	0,00	0,00	0,00	0,00
0,00	161,00	160,00	15,00	0,00	0,00	1,00	0,00
0,00	103,00	109,00	15,00	0,00	1,00	0,00	0,00
0,00	88,00	124,00	15,00	0,00	0,00	0,00	0,00
0,00	80,00	109,00	15,00	0,00	0,00	0,00	0,00
0,00	79,00	151,00	15,00	0,00	0,00	0,00	0,00
0,00	107,00	144,00	15,00	0,00	0,00	0,00	0,00
2,00	102,00	194,00	15,00	0,00	1,00	0,00	0,00
0,00	100,00	118,00	15,00	0,00	0,00	0,00	0,00
0,00	85,00	109,00	15,00	0,00	0,00	0,00	0,00
0,00	88,00	135,00	15,00	0,00	0,00	0,00	0,00
4,00	78,00	166,00	15,00	0,00	0,00	0,00	0,00
0,00	84,00	150,00	15,00	0,00	0,00	0,00	
5,00	79,00	192,00	11,00	0,00	0,00	0,00	0,00
			15,00	1,00	0,00	0,00	1,00
0,00	88,00	104,00	15,00	0,00	1,00	0,00	0,00
0,00	107,00	122,00	15,00	0,00	0,00	0,00	0,00
0,00	130,00	140,00	15,00	0,00	0,00	0,00	0,00
0,00	95,00	135,00	15,00	0,00	0,00	0,00	0,00
0,00	101,00	166,00	15,00	0,00	0,00	0,00	0,00

0,00	101,00	124,00	15,00	0,00	0,00	0,00	0,00
0,00	78,00	130,00	15,00	0,00	0,00	0,00	0,00
0,00	98,00	187,00	15,00	0,00	0,00	0,00	0,00
0,00	64,00	116,00	15,00	0,00	1,00	0,00	0,00
0,00	88,00	183,00	15,00	0,00	0,00	0,00	0,00
9,00	84,00	150,00	15,00	0,00	0,00	1,00	0,00
0,00	81,00	113,00	15,00	0,00	1,00	0,00	0,00
0,00	190,00	128,00	15,00	1,00	0,00	0,00	1,00
0,00	97,00	121,00	15,00	0,00	0,00	1,00	0,00
0,00	95,00	125,00	15,00	0,00	1,00	0,00	0,00
0,00	76,00	179,00	15,00	1,00	0,00	0,00	1,00
0,00	96,00	107,00	15,00	0,00	0,00	0,00	1,00
0,00	93,00	134,00	15,00	1,00	0,00	0,00	1,00
0,00	89,00	168,00	15,00	0,00	1,00	0,00	0,00
0,00	68,00	154,00	12,00	0,00	0,00	1,00	0,00
6,00	109,00	108,00	11,00	0,00	1,00	0,00	0,00
9,00	150,00	121,00	15,00	0,00	1,00	0,00	0,00
0,00	96,00	136,00	15,00	0,00	0,00	0,00	0,00
			15,00	0,00	0,00	1,00	0,00
0,00	98,00	131,00	15,00	0,00	0,00	1,00	
2,00	82,00	116,00	15,00	0,00	1,00	0,00	0,00
0,00	138,00	122,00	15,00	1,00	0,00	0,00	1,00
0,00	117,00	159,00	15,00	1,00	0,00	0,00	1,00
3,00	113,00	90,00	15,00	1,00	0,00	0,00	0,00
3,00	85,00	166,00	15,00	0,00	1,00	0,00	0,00
0,00	98,00	132,00	15,00	1,00	0,00	0,00	
0,00	90,00	139,00	15,00	0,00	1,00	0,00	0,00
5,00	97,00	131,00	15,00	0,00	1,00	0,00	0,00
0,00	72,00	140,00	14,00	0,00	1,00	0,00	0,00
0,00	96,00	171,00	15,00	0,00	0,00	0,00	0,00
0,00	75,00	156,00	15,00	0,00	1,00	0,00	0,00
0,00	104,00	134,00	15,00	0,00	0,00	0,00	0,00
2,00	95,00	146,00	15,00	0,00	0,00	0,00	0,00
0,00	110,00	171,00	15,00	0,00	0,00	1,00	0,00
2,00	75,00	91,00	13,00	0,00	1,00	0,00	0,00
0,00	120,00	142,00	15,00	0,00	0,00	0,00	
6,00	100,00	111,00	15,00	0,00	0,00	0,00	0,00
0,00	75,00	105,00	15,00	0,00	1,00	0,00	0,00
0,00	105,00	124,00	15,00	0,00	1,00	0,00	0,00
4,00	113,00	211,00	15,00	0,00	0,00	0,00	0,00
0,00	128,00	185,00	15,00	0,00	0,00	0,00	0,00
0,00	92,00	163,00	15,00	1,00	0,00	0,00	0,00
3,00	118,00	168,00	15,00	0,00	0,00	1,00	0,00
2,00	95,00	91,00	15,00	0,00	0,00	1,00	0,00
3,00	115,00	135,00	15,00	0,00	0,00	0,00	0,00
9,00	115,00	90,00	14,00	0,00	0,00	1,00	0,00
0,00	85,00	215,00	12,00	0,00	0,00	0,00	0,00
0,00	74,00	109,00	15,00	1,00	0,00	0,00	1,00
0,00	65,00	141,00	15,00	0,00	0,00	0,00	0,00
2,00	100,00	118,00	15,00	0,00	0,00	0,00	0,00

0,00	89,00	152,00	15,00	0,00	0,00	0,00	0,00
9,00	124,00	132,00	15,00	0,00	0,00	0,00	0,00
3,00	77,00	118,00	15,00	0,00	0,00	0,00	0,00
0,00	89,00	115,00	15,00	1,00	0,00	0,00	1,00
3,00	91,00	102,00	15,00	1,00	0,00	0,00	1,00
3,00	77,00	132,00	15,00	0,00	0,00	0,00	
0,00	89,00	164,00	15,00	0,00	0,00	0,00	0,00
0,00	78,00	201,00	15,00	1,00	0,00	0,00	1,00
0,00	75,00	122,00	15,00	0,00	0,00	0,00	0,00
4,00	120,00	125,00	15,00	1,00	0,00	0,00	1,00
3,00	101,00	195,00	15,00	1,00	0,00	0,00	1,00
9,00	76,00	83,00	13,00	0,00	1,00	0,00	0,00
9,00	105,00	149,00	15,00	0,00	0,00	1,00	
0,00	100,00	142,00	15,00	0,00	1,00	0,00	0,00
0,00	91,00	109,00	15,00	0,00	0,00	1,00	0,00
0,00	102,00	135,00	15,00	0,00	1,00	0,00	0,00
0,00		147,00	15,00	0,00	0,00	1,00	0,00
0,00	74,00	124,00	15,00	0,00	0,00	0,00	0,00
0,00	98,00	112,00	15,00	0,00	0,00	0,00	0,00
0,00	108,00	148,00	15,00	1,00	0,00	0,00	
0,00	96,00	127,00	15,00	0,00	0,00	0,00	0,00
0,00	101,00	144,00	15,00	1,00	0,00	0,00	1,00
0,00	64,00	124,00	15,00	0,00	0,00	0,00	0,00
0,00	97,00	149,00	15,00	1,00	0,00	0,00	1,00
0,00	100,00	126,00	15,00	0,00	0,00	1,00	0,00
0,00	71,00	121,00	15,00	0,00	0,00	0,00	0,00
2,00	164,00	166,00	15,00	0,00	0,00	0,00	0,00
0,00	74,00	147,00	15,00	0,00	1,00	0,00	0,00
0,00	90,00	162,00	15,00	0,00	0,00	0,00	0,00
0,00	112,00	152,00	15,00	1,00	0,00	0,00	1,00
0,00	89,00	157,00	15,00	0,00	1,00	0,00	0,00
0,00	86,00	166,00	15,00	0,00	0,00	0,00	0,00
0,00	76,00	136,00	15,00	0,00	0,00	1,00	0,00
0,00	86,00	126,00	15,00	0,00	0,00	1,00	0,00
0,00	78,00	155,00	15,00	0,00	1,00	0,00	0,00
0,00	82,00	160,00	15,00	0,00	0,00	0,00	0,00
0,00	71,00	125,00	15,00	0,00	0,00	0,00	0,00
0,00	98,00	155,00	15,00	0,00	0,00	0,00	0,00
0,00	107,00	112,00	15,00	0,00	0,00	0,00	0,00
0,00	86,00	137,00	15,00	0,00	1,00	0,00	0,00
3,00	102,00	154,00	15,00	0,00	0,00	1,00	0,00
0,00	69,00	125,00	15,00	0,00	0,00	0,00	0,00
0,00	111,00	129,00	15,00	0,00	0,00	0,00	0,00
0,00	86,00	145,00	15,00	0,00	0,00	1,00	0,00
0,00	85,00	155,00	15,00	0,00	1,00	0,00	0,00
0,00	70,00	108,00	15,00	0,00	1,00	0,00	0,00
4,00	94,00	103,00	15,00	0,00	0,00	0,00	0,00
0,00	115,00	114,00	15,00	0,00	0,00	1,00	0,00
0,00	125,00	165,00	15,00	1,00	0,00	0,00	1,00
9,00	84,00	135,00	15,00	0,00	0,00	0,00	0,00

0,00	109,00	106,00	15,00	0,00	1,00	0,00	
0,00	100,00	136,00	15,00	0,00	0,00	0,00	0,00
0,00	75,00	165,00	15,00	0,00	1,00	0,00	0,00
0,00	69,00	126,00	15,00	0,00	0,00	0,00	0,00
2,00	101,00	119,00	15,00	0,00	0,00	0,00	0,00
0,00	84,00	148,00	15,00	0,00	0,00	0,00	0,00
0,00	101,00	121,00	15,00	0,00	0,00	0,00	0,00
0,00	104,00	95,00	15,00	1,00	0,00	0,00	1,00
0,00	122,00	131,00	15,00	0,00	0,00	0,00	0,00
0,00	82,00	118,00	15,00	1,00	0,00	0,00	1,00
0,00	66,00	171,00	15,00	0,00	0,00	0,00	0,00
0,00	73,00	99,00	15,00	0,00	0,00	0,00	0,00
0,00	74,00	160,00	15,00	0,00	0,00	0,00	0,00
0,00	81,00	174,00	15,00	0,00	1,00	0,00	0,00
0,00	100,00	109,00	15,00	0,00	0,00	0,00	0,00
0,00	134,00	115,00	15,00	0,00	0,00	0,00	0,00
5,00	76,00	114,00	15,00	0,00	0,00	0,00	0,00
0,00	87,00	104,00	15,00	0,00	0,00	0,00	0,00
0,00	94,00	123,00	15,00	0,00	0,00	0,00	0,00
4,00		171,00	15,00	0,00	0,00	0,00	0,00
0,00	73,00	148,00	15,00	0,00	0,00	0,00	
0,00	86,00	194,00	15,00	0,00	1,00	0,00	0,00
0,00	95,00	174,00	15,00	0,00	0,00	0,00	0,00
4,00	66,00	186,00	15,00	0,00	0,00	1,00	0,00
0,00	98,00	169,00	15,00	0,00	1,00	0,00	
0,00	84,00	138,00	15,00	0,00	1,00	0,00	0,00
4,00	90,00	167,00	15,00	0,00	0,00	0,00	0,00
0,00	98,00	144,00	15,00	0,00	0,00	0,00	0,00
0,00	101,00	106,00	15,00	1,00	0,00	0,00	1,00
0,00	127,00	109,00	15,00	0,00	0,00	1,00	
0,00	96,00	115,00	15,00	1,00	0,00	0,00	1,00
0,00	76,00	131,00	15,00	0,00	1,00	0,00	0,00
0,00	103,00	136,00	15,00	0,00	1,00	0,00	
0,00	69,00	117,00	15,00	0,00	0,00	0,00	
9,00	93,00	165,00	15,00	1,00	0,00	0,00	1,00
2,00	126,00	138,00	15,00	0,00	0,00	1,00	0,00
0,00	70,00	113,00	15,00	0,00	0,00	0,00	0,00
0,00	77,00	126,00	15,00	0,00	0,00	0,00	0,00

lab_fluB	discharged	lenght_hosp	death	_patient_e	n_admission
0,00	1,00		0,00	68,00	8,00
0,00	1,00		0,00	70,00	8,00
1,00	1,00		0,00	61,00	9,00
1,00	0,00	9,00	0,00	57,00	9,00
0,00	1,00		0,00	49,00	9,00
0,00	0,00	13,00	0,00	64,00	9,00
0,00	1,00		0,00	45,00	10,00
0,00	1,00		0,00	59,00	7,00
0,00	0,00	15,00	0,00	53,00	9,00
1,00	0,00	7,00	0,00	52,00	9,00
0,00	1,00		0,00		13,00
1,00	0,00	5,00	0,00	54,00	7,00
1,00	0,00	15,00	0,00	52,00	7,00
0,00	0,00	6,00	1,00	61,00	7,00
0,00	1,00		0,00	63,00	7,00
0,00	1,00		0,00	60,00	7,00
0,00	0,00	5,00	0,00	55,00	7,00
0,00	1,00		0,00	60,00	8,00
0,00	1,00		0,00	45,00	11,00
1,00	1,00		0,00	45,00	8,00
0,00	1,00		0,00	27,00	4,00
0,00	0,00	5,00	0,00	43,00	4,00
0,00	0,00	3,00	0,00	44,00	6,00
1,00	1,00		0,00	31,00	6,00
0,00	0,00	4,00	1,00	43,00	12,00
0,00	1,00		0,00	30,00	12,00
0,00	1,00		0,00	43,00	12,00
1,00	1,00		0,00	38,00	10,00
0,00	1,00		0,00	36,00	10,00
0,00	1,00		0,00	36,00	10,00
0,00	0,00	5,00	0,00	36,00	11,00
1,00	1,00		0,00	39,00	11,00
0,00	1,00		0,00	53,00	11,00
0,00	1,00		0,00	44,00	11,00
0,00	0,00	1,00	0,00	45,00	6,00
0,00	1,00		0,00		7,00
1,00	0,00	8,00	0,00	47,00	7,00
0,00	0,00	4,00	0,00	44,00	8,00
0,00	0,00	11,00	0,00		10,00
0,00	1,00		0,00	60,00	11,00
0,00	0,00	3,00	0,00	44,00	11,00
	1,00		0,00	45,00	11,00
0,00	0,00	8,00	1,00	75,00	9,00
0,00	1,00		0,00	39,00	11,00
1,00	1,00		0,00	49,00	11,00
0,00	1,00		0,00	25,00	7,00
0,00	1,00		0,00	16,00	7,00
0,00	1,00		0,00	18,00	7,00
0,00	0,00	4,00	0,00	18,00	7,00

0,00	1,00		0,00	18,00	7,00
0,00	0,00	2,00	0,00	62,00	2,00
0,00	0,00	4,00	0,00	62,00	11,00
1,00	1,00		0,00	29,00	11,00
0,00	1,00		0,00	26,00	5,00
0,00	0,00	1,00	1,00	28,00	5,00
1,00	1,00		0,00	66,00	12,00
0,00	0,00	23,00	0,00	45,00	11,00
0,00	0,00	5,00	0,00	37,00	10,00
1,00	1,00		0,00	68,00	17,00
0,00	0,00	16,00	0,00	56,00	10,00
0,00	1,00		0,00		10,00
0,00	1,00		0,00		5,00
1,00	1,00		0,00	47,00	4,00
0,00	1,00		0,00	46,00	2,00
1,00	0,00	3,00	0,00	55,00	7,00
1,00	0,00	8,00	0,00	55,00	12,00
0,00	1,00		0,00	59,00	12,00
0,00	1,00		0,00	59,00	12,00
	1,00		0,00	46,00	10,00
1,00	0,00	9,00	0,00	63,00	10,00
0,00	0,00	4,00	0,00	55,00	11,00
0,00	1,00		0,00	31,00	7,00
0,00	1,00		0,00	36,00	4,00
1,00	1,00		0,00	60,00	2,00
0,00	1,00		0,00	46,00	6,00
1,00	1,00		0,00	67,00	12,00
1,00	0,00	3,00	1,00	60,00	12,00
1,00	0,00	4,00	0,00	53,00	12,00
0,00	1,00		0,00		12,00
1,00	1,00		0,00	71,00	10,00
0,00	1,00		0,00	57,00	12,00
0,00	1,00		0,00	69,00	16,00
0,00	0,00	9,00	0,00	59,00	12,00
1,00	0,00	12,00	1,00	58,00	5,00
	1,00		0,00	61,00	3,00
0,00	0,00	38,00	0,00	52,00	3,00
1,00	1,00		0,00		9,00
1,00	1,00		0,00		10,00
0,00	0,00	4,00	1,00	74,00	11,00
0,00	0,00	7,00	0,00	76,00	10,00
0,00	1,00		0,00	75,00	10,00
0,00	0,00	7,00	0,00	61,00	14,00
0,00	0,00	27,00	0,00		5,00
0,00	0,00	7,00	0,00	71,00	13,00
0,00	0,00	6,00	1,00	52,00	12,00
0,00	0,00	14,00	0,00		12,00
0,00	0,00	3,00	0,00	74,00	12,00
0,00	1,00		0,00	78,00	12,00
0,00	1,00		0,00	58,00	12,00

0,00	1,00		0,00		10,00
0,00	0,00	7,00	0,00	58,00	10,00
0,00	0,00	3,00	0,00		10,00
0,00	1,00		0,00	46,00	12,00
0,00	0,00	11,00	0,00		12,00
	0,00	6,00	0,00	84,00	8,00
0,00	0,00	15,00	0,00		8,00
0,00	1,00		0,00		8,00
0,00	1,00		0,00		5,00
0,00	0,00	3,00	0,00		6,00
0,00	0,00	2,00	0,00	42,00	2,00
1,00	0,00	0,00	1,00		2,00
	0,00	2,00	0,00		4,00
1,00	1,00		0,00	43,00	2,00
0,00	0,00	15,00	0,00	52,00	10,00
1,00	0,00	7,00	0,00	66,00	10,00
0,00	1,00		0,00	70,00	10,00
0,00	1,00		0,00	55,00	9,00
0,00	1,00		0,00	66,00	9,00
	1,00		0,00	57,00	7,00
0,00	1,00		0,00	64,00	9,00
0,00	1,00		0,00	70,00	12,00
0,00	0,00	8,00	0,00	62,00	12,00
0,00	1,00		0,00	64,00	12,00
0,00	0,00	24,00	0,00	64,00	12,00
0,00	1,00		0,00	44,00	6,00
0,00	0,00	2,00	0,00	30,00	8,00
1,00	1,00		0,00	28,00	2,00
0,00	0,00	1,00	0,00	30,00	4,00
0,00	1,00		0,00	32,00	4,00
1,00	1,00		0,00	34,00	10,00
0,00	0,00	13,00	0,00	64,00	10,00
0,00	1,00		0,00	50,00	10,00
0,00	1,00		0,00	48,00	6,00
1,00	1,00		0,00	47,00	6,00
0,00	1,00		0,00	64,00	6,00
0,00	0,00	6,00	0,00	48,00	6,00
0,00	1,00		0,00	53,00	10,00
0,00	1,00		0,00	64,00	10,00
1,00	0,00	4,00	0,00	57,00	9,00
0,00	0,00	4,00	0,00	51,00	9,00
0,00	0,00	35,00	0,00	50,00	8,00
0,00	1,00		0,00	51,00	13,00
0,00	0,00	8,00	0,00	51,00	13,00
1,00	1,00		0,00	56,00	13,00
1,00	1,00		0,00	58,00	8,00
1,00	0,00	19,00	0,00	49,00	10,00
0,00	0,00	6,00	0,00	52,00	4,00
0,00	1,00		0,00	41,00	4,00
0,00	0,00	4,00	0,00	42,00	7,00

	1,00		0,00	44,00	8,00
0,00	1,00		0,00		10,00
1,00	0,00	3,00	0,00	51,00	10,00
0,00	1,00		0,00	50,00	11,00
0,00	0,00	6,00	0,00		11,00
0,00	0,00	4,00	0,00	50,00	11,00
0,00	0,00	1,00	0,00	55,00	10,00
0,00	0,00	4,00	0,00		4,00
0,00	1,00		0,00	56,00	17,00
0,00	1,00		0,00	63,00	11,00
0,00	0,00	7,00	0,00	64,00	11,00
0,00	0,00	21,00	0,00	53,00	10,00
0,00	0,00	4,00	0,00	55,00	12,00
1,00	0,00	10,00	0,00	55,00	12,00
0,00	0,00	2,00	0,00	62,00	6,00
0,00	1,00		0,00	48,00	6,00
0,00	1,00		0,00	42,00	3,00
0,00	0,00	8,00	0,00	41,00	12,00
0,00	1,00		0,00	54,00	5,00
0,00	1,00		0,00	57,00	13,00
	1,00		0,00	59,00	13,00
1,00	1,00		0,00		10,00
0,00	1,00		0,00	46,00	7,00
0,00	0,00	3,00	0,00	45,00	7,00
	1,00		0,00	44,00	8,00
1,00	1,00		0,00	40,00	4,00
0,00	0,00	41,00	1,00	33,00	3,00
0,00	0,00	6,00	1,00	33,00	10,00
0,00	0,00	6,00	0,00	53,00	11,00
	0,00	9,00	0,00	51,00	11,00
0,00	1,00		0,00	50,00	9,00
1,00	1,00		0,00	47,00	11,00
	1,00		0,00	53,00	13,00
	1,00		0,00	56,00	13,00
0,00	0,00	6,00	0,00	72,00	5,00
0,00	0,00	3,00	0,00	43,00	12,00
0,00	0,00	4,00	0,00	53,00	17,00
0,00	1,00		0,00	54,00	11,00
